# Supplementary material for: NMR-Based Metabolomic Analysis on the Protective Effects of Apolipoprotein A-I Mimetic Peptide against Contrast Media-Induced Endothelial Dysfunction
Source: Molecules. 2021 Aug 24;26(17):5123. doi: 10.3390/molecules26175123 (PMC8433922; doi:10.3390/molecules26175123)
Supplement: Supplementary file 1 [file molecules-26-05123-s001.zip › molecules-1281028-supplementary.pdf]

Supplementary Materials

Figure S1.

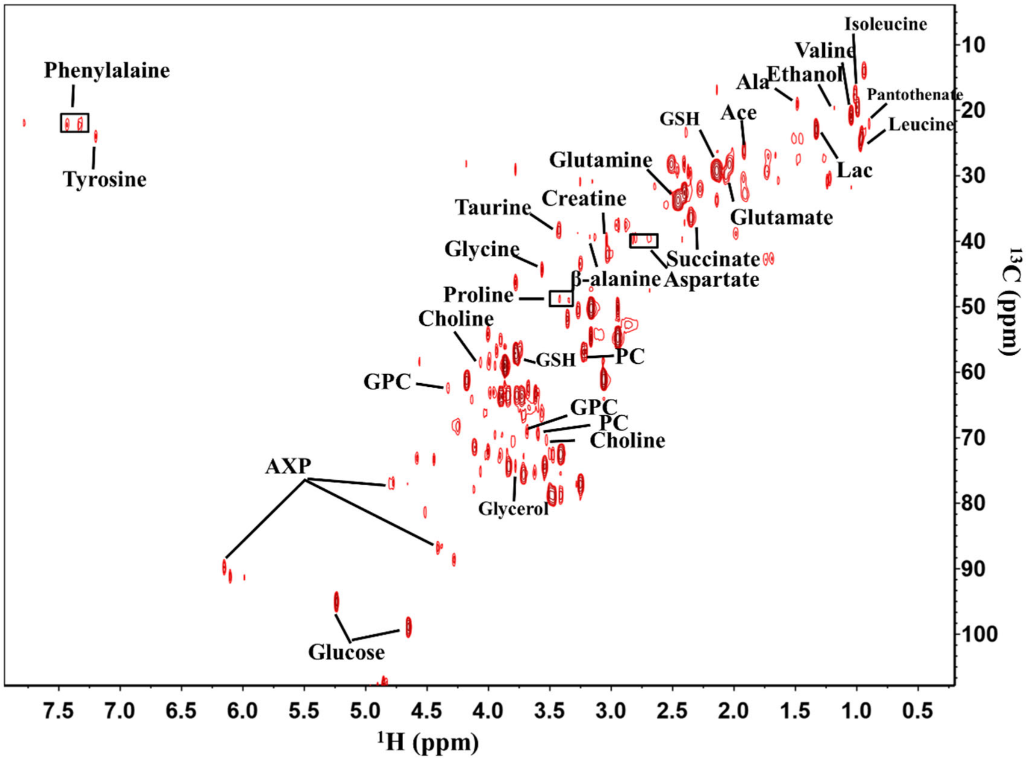

Figure S1. Typical 2D ^{1}H-^{13}C HSQC spectrum of aqueous extracts of HUVECs. The spectrum was recorded at 25 °C on a Bruker Advance III 850 MHz NMR spectrometer.

Figure S2.

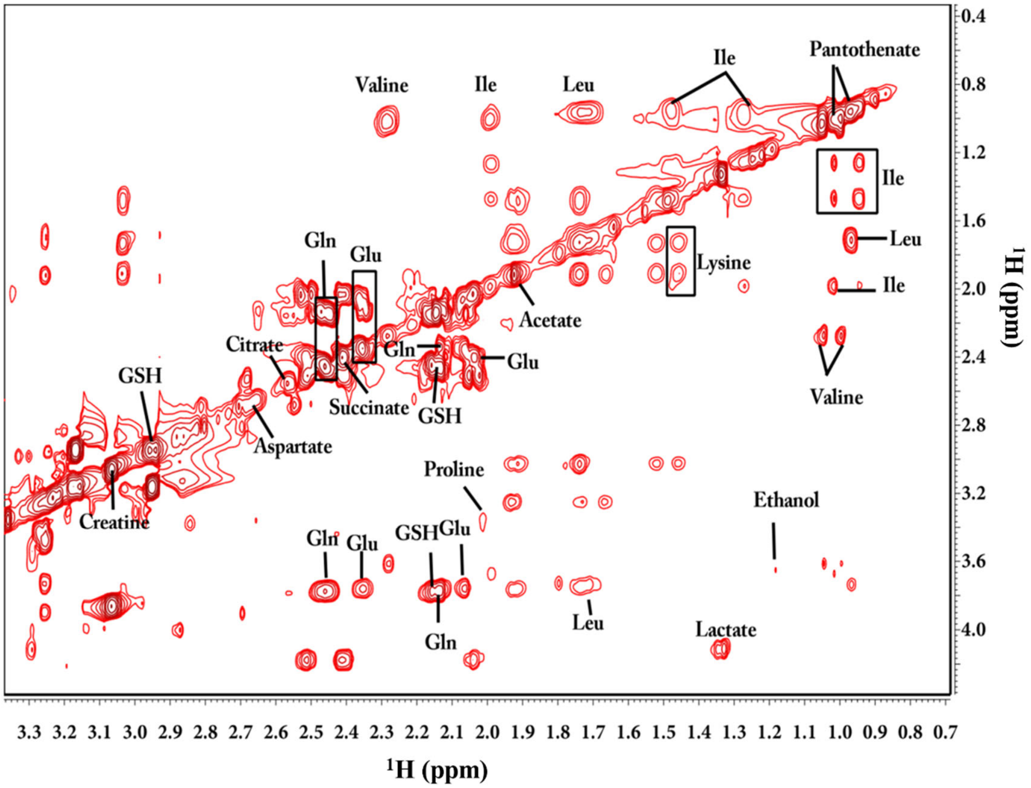

Figure S2. Region (0.7-3.3 ppm) of a typical 2D ^{1}H-^{1}H TOCSY spectrum of aqueous extracts of HUVECs. The spectrum was recorded at 25 °C on a Bruker Advance III 850 MHz NMR spectrometer.

Figure S3.

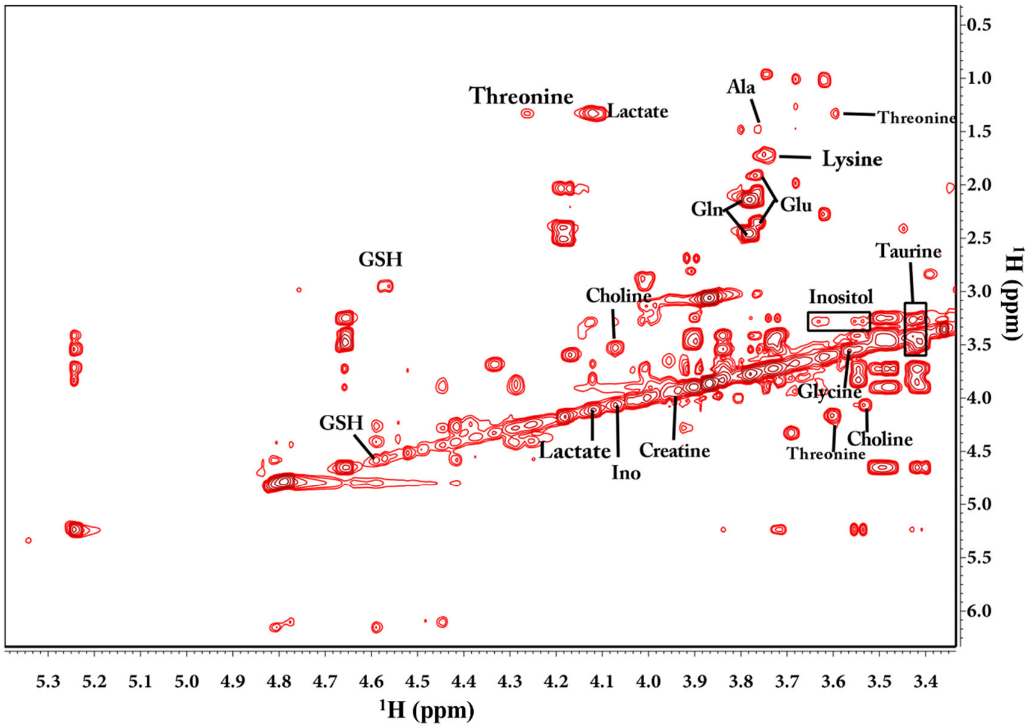

Figure S3. Region (3.4-5.3 ppm) of a typical 2D ^1H-^1H TOCSY spectrum of aqueous extracts of HUVECs. The spectrum was recorded at 25 °C on a Bruker Advance III 850 MHz NMR spectrometer.

Figure S4.

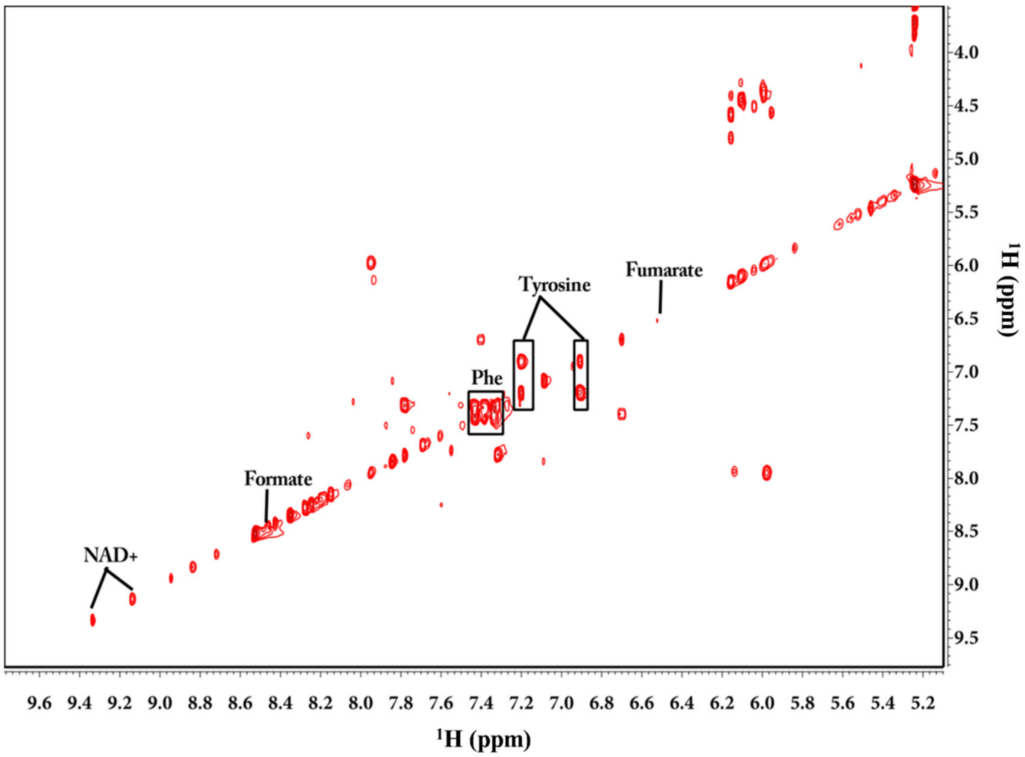

Figure S4. Region (5.2-9.6) ppm of a typical 2D ^{1}H-^{1}H TOCSY spectrum of aqueous extracts of HUVECs. The spectrum was recorded at 25 °C on a Bruker Advance III 850 MHz NMR spectrometer.

Figure S5.

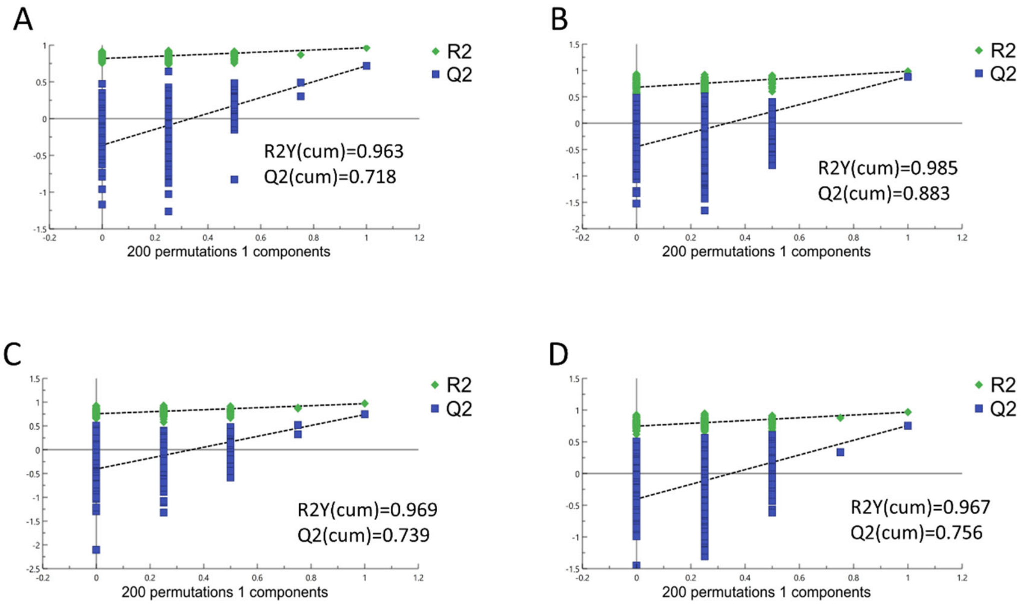

Figure S5. Cross-validation plots from response permutation tests for assessing reliabilities of the OPLS-DA models established for the five groups (Ctrl, Iod10, Iod30, D–4F+Iod10, D–4F+Iod30) of HUVECs (n=200). (A) Iod10 *vs*. Ctrl; (B) Iod30 *vs.* Ctrl; (C) D–4F+Iod10 *vs*. Iod10; (D) D–4F+Iod30 *vs*. Iod30.

Figure S6.

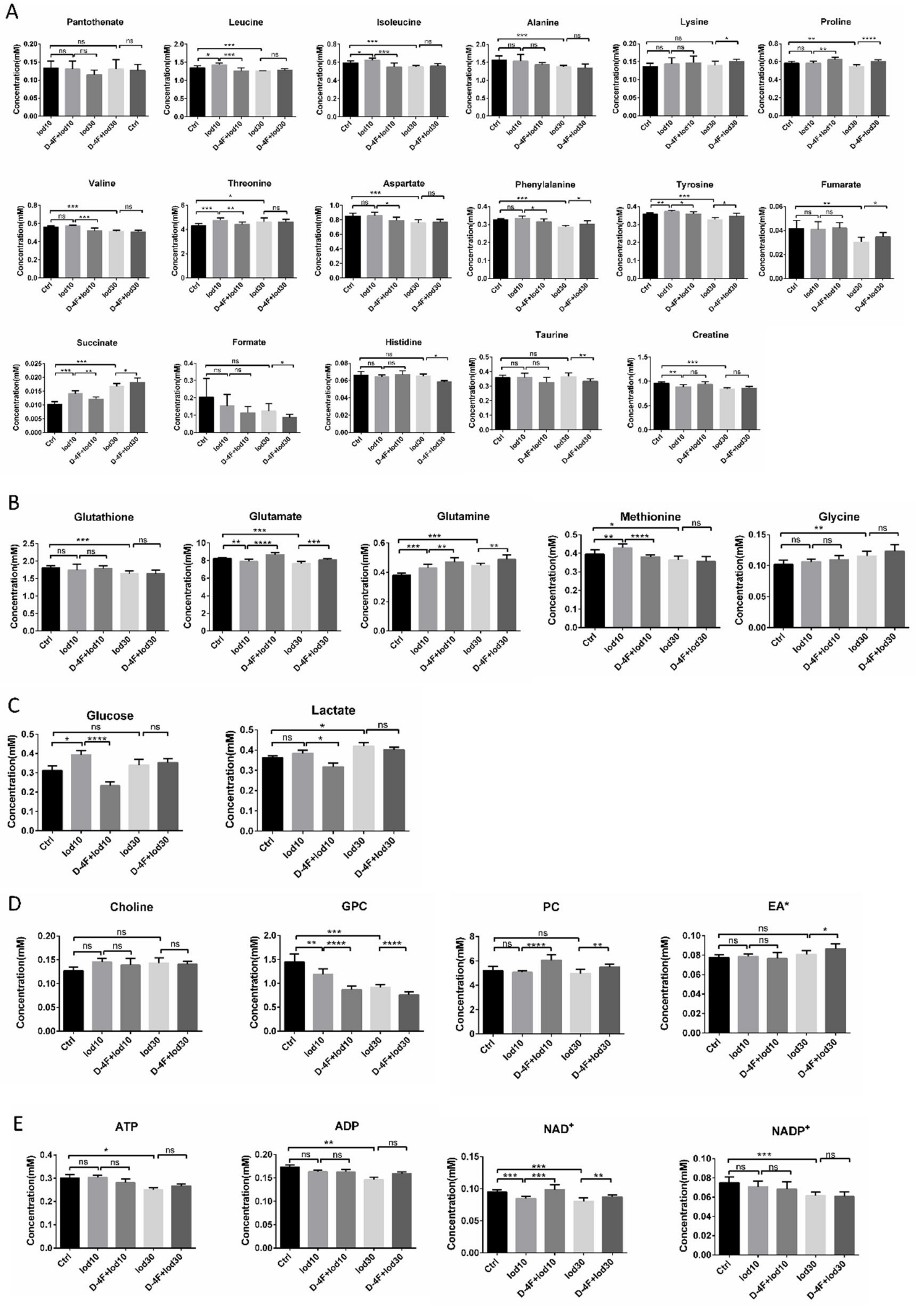

Figure S6. Pair-wise comparisons of absolute metabolite levels between the groups of HUVECs. (A) Comparisons of 17 metabolites related to amino acid metabolism (A), 5 metabolites related to glutathione metabolism (B), 2 metabolites related to glucose metabolism (C), 4 metabolites related to glycerophospholipid metabolism (D), other metabolites (E).

Figure S7.

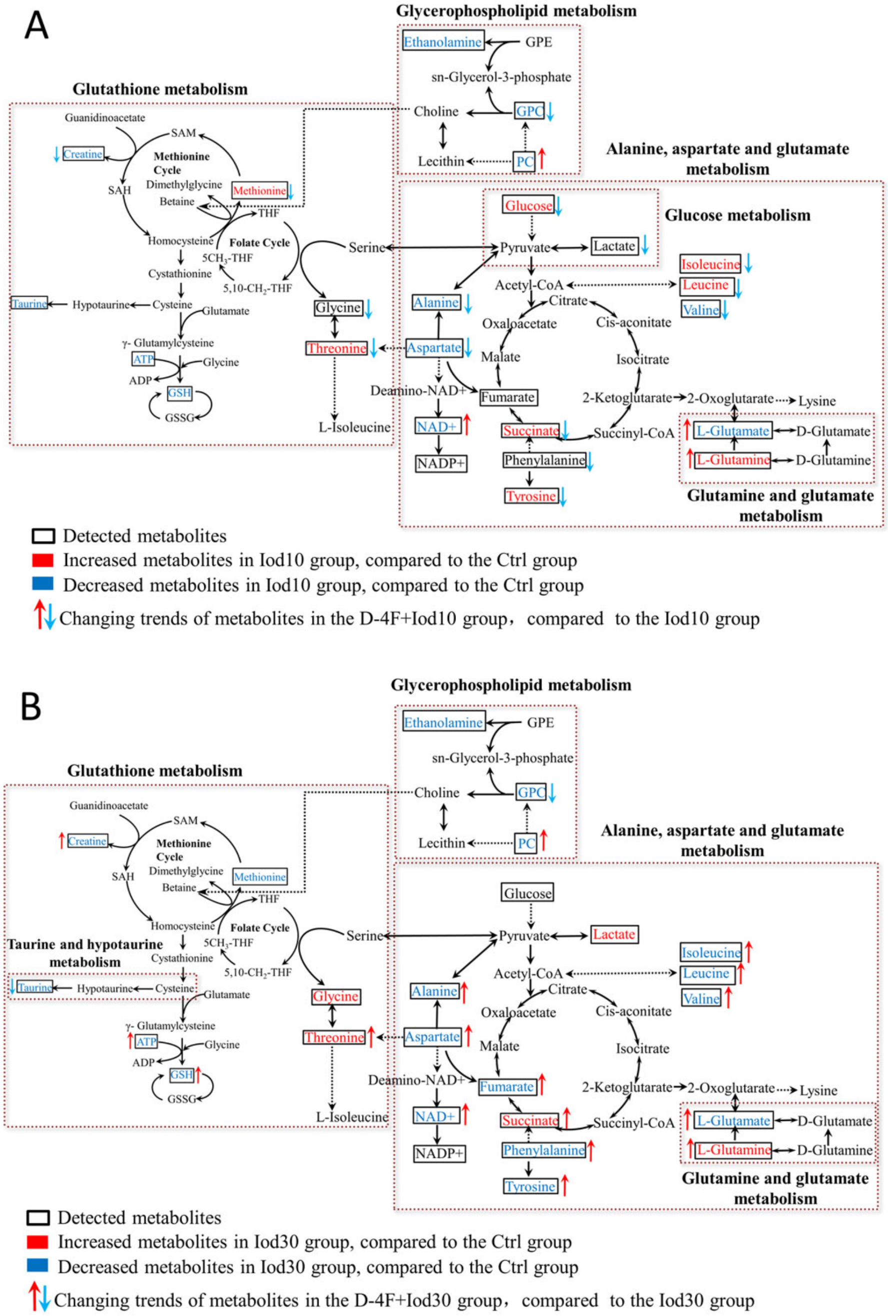

Figure S7. Schematic illustration of altered metabolic pathways in the D–4F+Iod10/D–4F+Iod30 groups compared with the Iod10/Iod30 groups. (A) Iod10 *vs*. Ctrl and D–4F+Iod10 *vs*. Iod10; (B) Iod30 *vs*. Ctrl and D–4F+Iod30 *vs*. Iod30.

Table S1. Resonance assignments of metabolites in 1D ^{1}H-NMR spectra of aqueous extracts of HUVECs.

NO.	Metabolite	δ ^{1}H (ppm) and multiplicy	Moieties#
1	Pantothenate	0.88(s),0.92(s)	CH_3, CH_3
2	Leucine	0.96(d),0.97(d),1.69(m), 1.70(m),1.73(m), 3.73(m)	α-CH_3, α-CH_3, γ-CH, β-CH_2, α-CH
3	Isoleucine	0.94(t),1.01(d),1.21(m), 1.42(m),2.00(m), 3.67(d)	δ-CH_3, γ-CH_3, half γ-CH_2, half γ-CH_2, β-CH, α-CH
4	Valine	0.99(d), 1.05(d), 2.26(m), 3.60(d)	γ-CH_3, γ-CH_3, β-CH, α-CH
5	Ethanol	1.17 (t), 3.65 (q)	δ-CH_3, CH_2
6	Threonine	1.30(d), 3.58(d), 4.24(m)	γ-CH_2, β-CH
7	Lactate	1.33(d), 4.11(q)	β-CH_3, α-CH
8	Alanine	1.47(d), 3.78(q)	β-CH_3,α-CH
9	Lysine	1.43(m),1.49(m),1.70(m), 1.91(m),3.02(t),3.75(t)	half γ-CH_2, half γ-CH_2, δ-CH_2, β-CH_2, ε-CH_2, α-CH
10	Acetate	1.91(s)	CH_3
11	Proline	1.99(m)	γ-CH_2
12	Glutamate	2.08(m),2.12(m),2.34(m), 2.37(m),3.75(m)	half β-CH_2, half β-CH_2, half γ-CH_2, half γ-CH_2, α-CH
13	Methionine	2.10(m),2.13(s),2.17(m), 2.66(t),3.78(m)	β-CH_2,SCH_3,β-CH_2,γ-CH_2,α-CH
14	Glutamine	2.13(m), 2.45(m), 3.77(t)	γ-CH_2, β-CH_2, α-CH
15	Succinate	2.42(s)	CH
16	Glutathione	2.15(m),2.55(m),2.96(m), 3.77(m),4.56(m)	β-CH_2, γ-CH_2, CH_2-SH,
17	Aspartate	2.69(dd),2.81(dd),3.90(dd)	β-CH_2,α-CH
18	DMA*	2.72(s)	CH_3
19	TMA*	2.83(s)	CH_3
20	Creatine	3.04(s), 3.93(s)	N-CH_3, α-CH_2
21	Choline	3.04(s), 3.93(s)	N-$(CH_3)_3$, α-CH_2,CH_2OH
22	PC*	3.22(s),3.60(t),4.18(m)	N-$(CH_3)_3$, α-CH_2,CH_2OH
23	GPC*	3.23(s),3.60(dd),3.68(dd),3.87(m),3.94(m),4.33(m)	N-$(CH_3)_3$, half $^1CH_2$,$^2CH_2$, half $^1CH_2$, half $^3CH_2$, half $^3CH_2$, $^1CH_2$
24	Taurine	3.27(t),3.43(t)	$^1CH_2$, $^2CH_2$
25	Glucose	β(3.24(dd), 3.48(t), 3.90(dd)), α(3.54(dd), 3.71(t), 3.72(dd), 3.83(m))	β(H_2, H_3, H_5), α(H_2, H_3, H_6)
26	EG*	3.72(s)	CH_2
27	EA*	3.14(m),3.83(t)	CH_2NH_2,CH_2OH
28	Glycine	3.57（s)	α-CH_2

29	Phenylalanine	3.12(dd),3.30(dd),3.99(dd),7.33(d),7.37(t),7.43(t）	α-CH, half β-CH_2,half β-CH_2,α-CH， β-CH,γ-CH
30	AXP*	6.14(d),8.27(s),8.58(s)	NH_2,δ-CH,^{2}CH
31	Histidine	7.06(s),7.85(s）	^{5}CH,^{2}CH
32	Fumarate	6.51(s)	CH
33	NAD^+	6.03(d),6.08(s),8.16(s), 8.20(m),8.41(s),8.82(d), 9.13(d),9.32(s)	NH_2,NH_2(CO),δ-CH,β-CH，^{2}CH,γ-CH,α-CH
34	$NADP^+$	6.05(d),6.15(d),8.16(s), 8.42(s),8.83(d),9.11(d), 9.29(s）	^{32}CH,^{2}CH,^{12}CH,^{7}CH,^{41}C,^{43}CH, ^{39}CH
35	Formate	8.46(s)	CH
36	Tyrosine	3.05(dd),3.19(dd),6.92(d),7.19(d)	half β-CH_2, half β-CH_2,β-CH,α-CH

Note: #: s, single; d, double; t, triplet; q, quartet; m, multiple; dd, double of double.

*: DMA, dimethylamine; TMA, trimethylamine; GPC, sn-glycero-3-phosphocholine; PC, O-phosphocholine; EG, ethylene glycol; EA, ethanolamine; AXP, ATP/ADP/AMP.

Table S2 Fold change values of absolute metabolite levels between the five groups of HUVECs.

Metabolite	Iod10 vs. Ctrl		D–4F+Iod10 vs.Iod10		Iod30 vs. Ctrl		D–4F+Iod30 vs.Iod30	
**Amino acid metabolism**								
Pantothenate	–	–	–	–	–	–	–	–
Leucine	1.06	↑	0.89	↓	0.93	↓	–	–
Isoleucine	–	–	0.88	↓	–	–	–	–
Valine	–	–	0.91	↓	–	–	–	–
Threonine	1.11	↑	0.93	↓	1.08	↑	–	–
Aspartate	–	–	–	–	0.89	↓	–	–
Alanine	–	–	–	–	0.88	↓	–	–
Lysine	–	–	–	–	–	–	1.08	↑
Proline	–	–	1.07	↑	–	–	1.10	↑
Phenylalanine	–	–	–	–	–	–	1.05	↑
Tyrosine	–	–	–	–	–	–	1.06	↑
Fumarate	–	–	–	–	–	–	1.14	↑
Formate	–	–	–	–	–	–	0.71	↓
Histidine	–	–	–	–	–	–	0.90	↓
Taurine	–	–	–	–	–	–	0.91	↓
Creatine	0.93	↓	–	–	0.88	↓	–	–
**Glutathione metabolism**								
Glutathione	–	–	–	–	0.90	↓	–	–
Glutamate	0.96	↓	1.10	↑	0.93	↓	1.06	↑
Glutamine	1.12	↑	1.10	↑	–	–	1.09	↑
Methionine	–	–	0.89	↓	–	–	–	–
**Glucose metabolism**								

Glucose	1.26	↑	0.59	↓	–	–	–	–
Lactate	–	–	–	–	–	–	–	–
**Glycerophospholipid metabolism**								
Choline	–	–	–	–	–	–	–	–
PC	0.97	↓	1.20	↑	0.96	↓	1.11	↑
GPC	0.82	↓	0.73	↓	0.63	↓	0.82	↓
EA	–	–	–	–	–	–	–	–
**Others**								
ATP	–	–	–	–	–	–	–	–
ADP	–	–	–	–	–	–	–	–
NAD+	–	–	1.16	↑	–	–	1.08	↑
NADP+	–	–	–	–	–	–	–	–

Note: ↑/↓: significantly increased/decreased levels of metabolites in Iod10 and Iod30 compared to Ctrl, D–4F+Iod10 compared to Iod10, D–4F+Iod30 compared to Iod30 with statistical significance $p < 0.05$.
